# Review of the Asthma Mortality Rate for Minnesota Residents Aged 55 Years or Older, 2004-2005: When Death Certificates Deserve a Second Look

**Published:** 2009-06-15

**Authors:** Wendy M. Brunner, Susan K. Ross, Jean E. S. Johnson

**Affiliations:** Minnesota Department of Health, Chronic Disease and Environmental Epidemiology; Minnesota Department of Health, Saint Paul, Minnesota; Minnesota Department of Health, Saint Paul, Minnesota

## Abstract

**Introduction:**

Asthma mortality rates are based on deaths for which asthma is coded as the underlying cause on the death certificate. We conducted an asthma mortality review to evaluate this surveillance measure for Minnesota residents who were aged 55 years or older.

**Methods:**

We enlisted an expert panel to review transcribed interviews from the next-of-kin and case histories for decedents whose deaths were attributed to asthma. In addition, we examined death certificates to determine whether the certifier had intended asthma to be the underlying cause.

**Results:**

In the age group of Minnesotans we examined, 55 deaths were attributed to asthma during the 1-year study period. Of the 35 deaths for which adequate information was available for review, 2 were determined to be due to asthma. On 33 of the 55 death certificates, the certifier had chosen asthma as the underlying cause; on the rest, the certifier had not chosen asthma, but inconsistencies in death certificate completion had resulted in "asthma" automatically overriding the underlying cause that had been chosen.

**Conclusion:**

Asthma mortality rates for older Minnesotans may be overestimated because of inaccurate reporting of the underlying cause of death on death certificates.

## Introduction

According to the Centers for Disease Control and Prevention (CDC) guidelines for asthma surveillance and the specifications for the *Healthy People 2010* asthma mortality indicator, asthma mortality rates should be calculated based on the number of deaths for which asthma is the underlying cause (1). During the development of a strategic plan for addressing asthma in Minnesota in 2002, advisory group members observed that asthma mortality rates in the state were highest among residents who were aged 65 or older and that the rate in this age group was twice as high as that for the same age group nationally. Noting the possibility of misdiagnosis of asthma in this age group, and the limitations of death certificates, the advisory group proposed that many of these deaths were not due to asthma and recommended that an asthma mortality review be conducted (2).

The purpose of this study was to evaluate the validity of the coding of asthma as the underlying cause of death in records used by the Minnesota Department of Health (MDH) Asthma Program to track asthma mortality. Inaccurate listing of cause of death affects mortality statistics and has implications for programs that rely on surveillance data to set priorities.

## Methods

We obtained death records from the MDH Office of the State Registrar for Minnesota residents aged 55 or older who died between July 1, 2004, and June 30, 2005, and whose underlying cause of death was coded as asthma (International Classification of Diseases, 10th revision [ICD-10] code J45 [asthma] or J46 [status asthmaticus]). Age 55 was chosen as the cutoff because state surveillance data show that the number of asthma deaths begins to rise at that age.

We attempted to locate the address and telephone number of the next-of-kin listed on each death certificate. We sent an introductory letter to the next-of-kin requesting a telephone interview, which included detailed questions about the deceased's medical history, smoking history, health care access, and circumstances of death. Next-of-kin who declined to be interviewed were asked to complete an abbreviated interview. After the interview, we asked for the next-of-kin's consent to release to MDH the deceased's medical records from a maximum of 2 years before death.

The MDH Asthma Program's registered nurse/certified asthma educator reviewed each record and interview for decedents who had 1 or both and wrote a case history for each decedent. We recruited a panel of 4 pulmonologists to review the case histories and determine both the likelihood that the decedents had asthma (definitely yes, probably yes, unable to determine, probably not, definitely not) and that asthma was the cause of death (yes, unable to determine, definitely not). Each case was presented by the primary reviewer and discussed by the panel. Differences between panelist determinations were resolved by consensus.

We also reviewed the cause-of-death section of the death certificates for all of the decedents, in consultation with the state nosologist. We examined whether the certifier (ie, physician or medical examiner/coroner) had intended asthma to be listed as the underlying cause of death and compared the death certificate reporting with the panel determinations regarding the underlying cause of death.

In Part I of the cause-of-death section of the death certificate, the certifier lists the sequence of conditions leading to the death; the immediate cause is listed first and the underlying cause is listed last. Other conditions that contributed to the death, but not in the causal sequence, are listed in Part II (3). Death certificates are then coded for consistency by state vital statistics offices and the National Center for Health Statistics according to the latest version of ICD. Discrepancies are resolved according to World Health Organization (WHO) rules for coding the underlying cause of death, which consider the reported duration of each condition listed and whether each condition listed could give rise to the condition listed above it in the sequence.

To determine whether the pattern of reporting was unique to the year we selected, we reviewed death certificates for asthma-coded deaths in people aged 55 or older from the previous year (July 1, 2003, through June 30, 2004). To determine whether the pattern of reporting was unique to the older age group, we reviewed death certificates for asthma-coded deaths from July 1, 2004, through June 30, 2005, for people who were younger than 55.

We used the Fisher exact test to compare proportions and the *t* test to compare means. This study was approved by the Minnesota Department of Health institutional review board.

## Results

Between July 1, 2004, and June 30, 2005, asthma was coded as the underlying cause of death for 55 Minnesota residents who were aged 55 or older. Age at death ranged from 61 to 103 years, and the median age was 85 (Table 1).

We were able to conduct an interview, obtain medical records, or both for 44 decedents (Table 2). Most next-of-kin interviewed were children of the decedents (n = 26), followed by spouses (n = 9), siblings (n = 3), unrelated caregivers (n = 2), and other relatives (n = 1).

Forty-four decedents (80%) were eligible for panel review (ie, had an interview, medical records, or both). Decedents who were not reviewed (next-of-kin not located, next-of-kin refused, or medical records not received for cases without a next-of-kin interview) were more likely to have been residents of the Twin Cities metropolitan area (*P* = .02) and ethnic minorities (*P* = .04). Differences by age group (55 to 79 years vs ≥80) (*P* = .51) and sex (*P* = .76) were not significant at the *P* = .10 level. Too few cases by category were available to compare by place of death.

Of the 44 reviewed decedents, the panel of pulmonologists determined that 11 probably or definitely had asthma, and 18 probably or definitely did not have asthma (Table 3). Among the 35 reviewed decedents for whom adequate records were available to decide whether asthma was the underlying cause of death, only 2 were determined to have died from asthma. For 5 decedents, the panel determined that the cause of death was unknown, and for 28 the panel determined that asthma was definitely not the cause of death. The panel was unable to make a determination for 9 of the deaths due to a lack of information (ie, incomplete medical records). Only 2 of the 55 decedents had had autopsies.

The finding of only 2 asthma deaths among the 44 reviewed led us to examine the death certificates for all of the decedents (N = 55) to determine why so few deaths were judged to be due to asthma by our panel. We found that in 33 of the 55 cases (60%), the certifier selected asthma as the underlying cause of the death by listing it last in the Part I series. The panel of pulmonologists reviewed 27 of the 33 cases and agreed with the certifier's assessment in only 2 of these cases (7%). In 9 of the 27 cases, the panel was unable to determine whether asthma was the underlying cause of death (7 because of incomplete records) and for 16, it determined that the cause of death was definitely not asthma.

In 22 of the 55 cases (40%), the certifier did not select asthma as the underlying cause of death, instead listing it elsewhere on the death certificate (all of the 55 death certificates had asthma listed either in Part I or II). Because of inconsistencies in the reporting, the WHO rules overruled the certifier's choice, requiring that asthma be listed as the underlying cause of death. In 18 cases, asthma was listed in Part II, but was moved to Part I as the underlying cause of death because the condition selected by the certifier as the underlying cause was ill-defined. The most common conditions that were overruled were "natural causes," "pneumonia," and "cardiopulmonary arrest." In 4 cases, the Part I sequence was considered illogical, resulting in the selection of asthma as the underlying cause. For example, on 1 death certificate, asthma was listed as a consequence of dementia, which is not considered a logical sequence under the WHO rules, and asthma was selected as the underlying cause of death.

Decedents for whom the certifier selected asthma as the underlying cause did not differ from those for whom the certifier selected another condition as the underlying cause by age (*P* = .40), sex (*P* = .53), residence (*P* = .61), or whether they had undergone panel review (*P* = .47) (Table 4). Too few cases by category were available to compare by place of death.

In 24 of the 41 cases for which a next-of-kin interview was conducted, the deceased was reported to have been a current or former smoker. In 16 of the 25 reviewed cases in which the certifier had selected asthma, the decedent was a current or former smoker, compared with 8 of the 16 reviewed cases in which the certifier had not selected asthma.

The findings were similar among the 46 asthma-coded deaths in people 55 or older in the previous year: on 25 (54%) of the death certificates, asthma was the intended underlying cause of death, while on 21 (46%), asthma overrode the reported cause because of the WHO rules. Among the 15 decedents younger than 55 who died during the study period, asthma was the intended underlying cause of death in 13 (87%) of the cases, and the reported cause was overruled in only 2 (13%).

## Discussion

Previous studies have shown that death certificates may underestimate (4) or overestimate (5-8) the true number of asthma deaths in a population. Most of these studies examined the deaths of people who had been hospitalized for asthma or were otherwise known to have asthma. In a population-based study that followed people with asthma or chronic obstructive pulmonary disease (COPD), researchers reported a false-negative rate of 58%. Interestingly, they did not find any death certificates coded as asthma outside of their asthma/COPD cohort (4). In another study of the false-negative rate, the authors found that asthma deaths tended to be misclassified as cardiovascular disease or COPD (6).

In contrast, our study assessed the validity of an existing asthma mortality measure by focusing on the false-positive rate. In a similar population-based study conducted in Denmark in all age groups, the authors examined deaths coded as asthma and found that the accuracy of Danish death certification in asthma deaths was poor, especially in the elderly, where COPD was often classified as asthma (8).

We found that death certificates with asthma coded as the underlying cause of death overestimated asthma mortality among Minnesotans aged 55 or older. The reasons for the observed false-positives fell into 2 categories: 1) the certifier incorrectly selected asthma as the underlying cause of death (choice differed from panel’s) because of the patient’s age at death or comorbidities or because the certifier was not the patient’s primary care provider or 2) the certifier did not select asthma as the underlying cause of death, but the WHO rules for coding the underlying cause overrode the original selection because of an illogical sequence or selection of a condition not eligible to be an underlying cause of death (eg, “natural causes”). Generally, if the condition listed last in Part I was an ineligible underlying cause and asthma was listed next in Part I or first in Part II, asthma was selected by the computer algorithm as the underlying cause.

COPD is often misdiagnosed as asthma in people older than 40 (9). We found evidence that the presence of COPD contributed to the difficulty of accurately determining the underlying cause of death. Among the 28 death certificates that listed another condition above asthma in the Part I sequence, 12 listed COPD. In addition, decedents for whom the certifier incorrectly selected asthma as the underlying cause of death were more likely to be have been current or former smokers.

Previous studies have found that the percentage of false-positives for asthma as the cause of death increases with age (6,7). Most decedents in our study were 80 or older when they died. More than half of those for whom the certifier selected asthma as underlying cause died in a nursing home.

On the basis of our findings, only 2 deaths among Minnesota residents aged 55 or older during the 1-year study period could be verified as being caused by asthma. Even if the 20 cases for which the panel was unable to determine the cause of death because of lack of information were categorized as asthma deaths, the resulting asthma mortality rate would still be less than half the observed rate (based on 22 deaths rather than 55). This finding puts Minnesota much closer to or under the national rate, although other states' patterns of reporting may also be prone to inconsistencies. However, Minnesota residents have the second highest life expectancy in the United States, behind Hawaii (10,11), so if these trends in filling out death certificates are related to the age of the decedent, then Minnesota (and Hawaii) may be different. Our study did not examine deaths attributed to other causes to see how many may actually have been asthma, so the true rate of asthma mortality among older Minnesotans is unknown.

If Minnesota's experience is representative, the false-positive rate for asthma mortality among older populations is high. On the basis of these findings, we suggest that 1) when conducting asthma surveillance, states examine death certificates for residents aged 55 or older for whom the underlying cause of death was coded as asthma to determine the extent of miscoding (ie, the percentage of certificates that do not list asthma last in the Part I sequence); 2) because of the difficulty in distinguishing asthma and COPD in older people, chronic lower respiratory disease — a category that includes asthma, COPD, and bronchiectasis — may be a more useful measure for surveillance; and 3) further research should be done into the false-negative rate.

This study was limited by the amount of information we could obtain for each decedent. We were unable to locate the next-of-kin in 6 cases. There is no way to know if the decedents whose death certificates were not reviewed by the panel were different from those for whom complete information was available in terms of accuracy of the reported underlying cause of death. The medical records we received were frequently incomplete, and information provided by the next-of-kin varied greatly depending on the next-of-kin's level of involvement in the decedent's care.

Asthma mortality rates among Minnesota residents aged 55 or older may be inflated because of inaccurate and improper reporting of causes of death on the death certificate. These findings have implications for asthma surveillance and for studies using vital records to track asthma mortality.

## Acknowledgments

This work was supported by CDC cooperative agreement no. 2U59EH522470-06. We thank the interviewers for this study: Jessica Lundin, Joanne Moze, and Margee Brown; the members of our review panel: Drs Krista Graven, Melissa King-Biggs, Charlene McEvoy, and Jeff Rubins; and MDH nosologist Diane Dalin. We especially thank the next-of-kin whose participation made this study possible.

## Figures and Tables

**Table 1:** Characteristics of Minnesota Residents Aged 55 or Older Whose Underlying Cause of Death Was Coded as Asthma (N = 55), July 1, 2004, through June 30, 2005

**Characteristic**	n (%)
**Age, y**	
55-59	0
60-69	10 (18.1)
70-79	8 (14.5)
80-89	18 (32.7)
≥90	19 (34.5)
**Sex**	
Female	44 (80.0)
Male	11 (20.0)
**Race/ethnicity**	
White	51 (92.7)
Other	4 (7.3)
**Place of death**	
Nursing home	26 (47.3)
Hospital inpatient	18 (32.7)
Residence	8 (14.5)
Outpatient/emergency department/other	3 (5.5)
**Residence at death**	
Greater Minnesota	30 (54.5)
Twin Cities metropolitan area^a^	25 (45.5)

a The 7-county Twin Cities metropolitan area, comprising Minneapolis and Saint Paul as well as the counties of Anoka, Carver, Dakota, Hennepin, Ramsey, Scott, and Washington.

**Table 2:** Process for Asthma Mortality Review for Minnesota Residents Aged 55 or Older Whose Underlying Cause of Death Was Coded as Asthma, July 1, 2004, through June 30, 2005

Asthma Deaths	Death Certificates Obtained	Next-of-Kin Located	Next-of-Kin Interview Conducted	Medical Records Obtained	Eligible for Panel Review
N = 55	N = 55	Yes (n = 49)	Yes (n = 36)	Yes (n = 31)	Yes (n = 44)
				No (n = 5)	
			Yes, abbreviated (n = 5)	Yes (n = 2)	
				No (n = 3)	
			No (n = 8)	Yes (n = 3)	
				No (n = 5)	No (n = 11)
		No (n = 6)	NA	NA	

Abbreviation: NA, not applicable.

**Table 3:** Determinations Regarding Asthma Status and Cause of Death for 44 Reviewed Cases Among Minnesota Residents Aged 55 or Older Whose Underlying Cause of Death Was Coded as Asthma (N = 55), July 1, 2004, through June 30, 2005^a^

**Characteristic**	n (%)
**Had asthma**	
Definitely yes	4 (9.1)
Probably yes	7 (15.9)
Unable to determine	15 (34.1)
Probably no	9 (20.5)
Definitely no	9 (20.5)
**Died of asthma**	
Yes	2 (4.5)
Unable to determine (complete records)	5 (11.4)
Unable to determine (lack of information)	9 (20.5)
Definitely no	28 (63.6)

a Although asthma was coded as the underlying cause for 55 deaths, medical records, next-of-kin interviews, or both were available for only 44 decedents.

**Table 4:** Characteristics of Decedents for Whom Asthma Was Listed as the Underlying Cause of Death on the Death Certificate, Minnesota Residents Aged 55 or Older (N = 55), July 1, 2004, through June 30, 2005

**Characteristic**	Asthma Listed as Underlying Cause of Death on Death Certificate	
	Yes (n = 33)	No (n = 22)
**Mean age, y**	82	85
**Age range, y**	61-97	64-103
**Female sex, %**	78.8	81.8
**Place of death, %**		
Nursing home	54.5	36.4
Hospital inpatient	36.4	27.3
Emergency department/other/outpatient	3.0	9.1
Residence	6.1	27.3
**Residence at death, %**		
Greater Minnesota	54.5	54.5
Twin Cities metropolitan area^a^	45.5	45.5
Reviewed by panel, %	81.8	77.3

a The 7-county Twin Cities metropolitan area, comprising Minneapolis and Saint Paul as well as the counties of Anoka, Carver, Dakota, Hennepin, Ramsey, Scott, and Washington.
